# A health state utility valuation study to assess the impact of treatment mode of administration in Gaucher disease

**DOI:** 10.1186/s13023-018-0903-6

**Published:** 2018-09-10

**Authors:** Monica Hadi, Paul Swinburn, Luba Nalysnyk, Alaa Hamed, Atul Mehta

**Affiliations:** 1 Patient-Centered Outcomes, Mapi, London, UK; 2 Sanofi Genzyme, 50 Binney Street, Cambridge, MA, 02142 USA; 3 Lysosomal Storage Disorders Unit, Department of Haematology, Royal Free Hospital and University College Medical School, London, UK

**Keywords:** Utility, United Kingdom, Gaucher disease, Treatment mode of administration

## Abstract

**Background:**

This study aimed to obtain UK societal-based utility values for health states related to treatment mode of administration using Gaucher disease as the background condition.

**Methods:**

A review of relevant literature and expert clinical input informed the development of five health states characterising the impact of Gaucher disease and its management on patients’ lives. A base-state characterising the “controlled disease” was developed as well as four subsequent health states which varied in description of the method (intravenous versus oral) and frequency of treatment administration. Health state utilities were obtained using the time trade-off (TTO) method via face-to-face interviews with 100 members from the UK general population. Before the valuation exercise, participants provided informed consent, completed a demographic form and the EQ-5D, and ranked the health states from best to worst on a 0–100 visual analogue scale (VAS).

**Results:**

Mean age of the participants (*n* = 100) was 35 years and 66% were female. Participants reported high EQ-5D VAS (86.1) and index scores (0.95) indicating very good health status. The “controlled disease” state had the highest mean TTO-derived utility value (0.89). There was only a marginal reduction in utility for the generic state for “Oral treatment” (0.85), while the reduction was more pronounced for the generic state for “Intravenous treatment” (0.73).

**Conclusions:**

The findings suggest that the avoidance of the need for intravenous treatment administration is associated with a notable positive increase in health-related quality of life. Patient benefit arising from less invasive treatment could be an important consideration when undertaking economic evaluation of future therapies for Gaucher disease.

**Electronic supplementary material:**

The online version of this article (10.1186/s13023-018-0903-6) contains supplementary material, which is available to authorized users.

## Background

Over the past decades, treatments with similar efficacy have been developed in several indications with the main differences being in the mode of administration, intravenous infusion, subcutaneous injection, oral intake [1, 2]. Gaucher disease, a rare, genetic lysosomal storage disorder caused by a deficiency in the enzyme acid beta-glucosidase, is an example of a disease for which treatment exists with different modes of administration; intravenously-administered enzyme replacement therapy and orally-administered substrate reduction therapy. Gaucher disease affects patients of all ages and is one of the most common lysosomal storage diseases with a worldwide prevalence of approximately 1/100,000 and approximately 1/855 in the Ashkenazi Jewish population [3, 4]. There are three commonly recognised types of Gaucher disease. Type 1 is the most common form of Gaucher disease in the United States and Europe. It is associated with debilitating visceral, haematological and skeletal manifestations characterized by liver and/or spleen enlargement, anaemia, fatigue, thrombocytopenia leading to easy bruising and bleeding episodes, and debilitating bone pain and bone fractures [5]. The two more severe forms of Gaucher disease, type 2 which manifests in early infancy and type 3 which manifests in early childhood, are associated in addition with overt neurological symptoms [6].

There is currently no cure for this disease. The two treatment options for Gaucher disease type 1 (GD1) that exist aim at reducing toxic accumulation of the substrate glucosylceramide and other glycolipids, subsequently preventing progressive disease with debilitating complications [7]. The two options are enzyme replacement therapy (ERT) (imiglucerase [8], velaglucerase alfa [9] and taliglucerase alfa [10]) and substrate reduction therapy (SRT) (eliglustat [11, 12] and miglustat [13]). Both ERTs and SRTs require lifelong treatment. ERTs are administered intravenously, usually every two weeks in an outpatient procedure. The SRT eliglustat was recently approved by both the US Food and Drug Administration (FDA) in 2014 [14] and the European Medicines Agency (EMA) in 2015 [15] as a first line treatment in previously treated and treatment-naive adults with GD1 who have compatible CYP2D6 metaboliser phenotypes (> 90% of patients) [16, 17]. The SRT miglustat is also approved by FDA and EMA as a second-line treatment for adults with GD1 who cannot be treated with ERT [18, 19]. SRT is typically administered orally on daily basis.

Intravenously-administered ERT imiglucerase and orally-administered SRT eliglustat have demonstrated comparable efficacy [11], however, the value of oral versus intravenous treatment in relation to patient’s benefit, compliance, preference and quality of life has not been investigated in the context of Gaucher disease and only been poorly investigated in other conditions. Two reviews in diabetes have shown that efficacy and potential side effects played an important role in determining patient satisfaction which outweighed aspects linked to the mode of administration [20, 21]. In the context of iron overload in patients with beta-thalassaemia, the available orally-administered treatment was preferred over the subcutaneously-administered treatment since it was found to be more convenient, did not involve injection-site soreness, and was less disruptive to engaging in usual day activities, sleep patterns and family life [22].

Most health technology assessment (HTA) bodies require cost-utility analysis to be performed to feed medico-economic assessment when health-related quality of life is an important consequence of the studied intervention. Utility values represent the strength of an individual’s preferences for specific health-related outcomes [23]. A utility value of 1 represents perfect health and a utility value of 0 represents a state equivalent to dead; a negative value represents a state worse than death.

The present study aimed to use a time-trade-off (TTO) vignette-based approach [24] to estimate the utility values associated with each health state related to treatment mode of administration in GD1 as the background condition.

## Methods

### Health state development

In order to inform the development of the health state descriptions, a review of published literature describing available therapies and of the corresponding product labels (i.e. imiglucerase and eliglustat) was undertaken. In total, five health states were developed. First, a benchmark state was described as “controlled disease” in which a life-long treatment is taken on a regular basis. Then, two generic mode of administration health states were developed for intravenously-administered treatment (life-long treatment administered as intravenous infusions every 2 weeks with the possibility of experiencing infusion-related reaction) and orally-administered treatment (life-long treatment administered orally every day with the possibility of experiencing minor treatment-related side-effects). Two alternative health states for the orally-administered treatment were developed, more closely mirroring current treatment options (alternative 1 with a reduced frequency of intake and side effects; alternative 2 with a higher frequency of intake and more frequent occurrence of side effects). In the development of the health states for this study, it was assumed that treatments had comparable efficacy.

Next, to validate the draft health state descriptions, an interview was undertaken with a clinician (AM) who had extensive expertise of managing patients with Gaucher disease. The interview involved working through each of the health state descriptions and assessing the extent to which they were both representative and accurate depictions of the intended treatment scenarios.

Lastly, prior to developing the final heath state descriptions, a piloting exercise via cognitive debriefing was undertaken with a small sample of members of the UK general public (*n* = 6). Participants were asked to read through the various descriptions carefully and then were asked a series of questions to ensure that the description wording used was well-understood. After a few minor revisions, the health states were finalized. Table 1 lists the generic mode of administration health states; the full set of health state descriptions is available in Additional file 1: Table S1.

**Table 1:** Health state descriptions for the generic states

Health states	Descriptions
Controlled disease	• You have an inherited condition that may lead to you developing health problems. These problems could include tiredness, issues with your bones causing pain and becoming more likely to fracture, and enlargement of your liver and spleen which can result in serious complications. • In order to try and prevent problems developing you are required to receive treatment for the rest of your life. The treatment is effective as long as it is taken according to instructions. • You need to take the treatment on a regular basis. You need to consider your access to treatment when travelling.
Intravenous treatment	• You have an inherited condition that may lead to you developing health problems. These problems could include tiredness, issues with your bones causing pain and becoming more likely to fracture, and enlargement of your liver and spleen which can result in serious complications. • In order to try and prevent problems developing you are required to receive treatment for the rest of your life. The treatment is effective as long as it is taken according to instructions. • The treatment you need to take is administered intravenously. You need to receive a 1 to 2-h infusion (directly into a vein) every 2 weeks. You also need to consider your access to treatment when travelling as the infusion must be administered by, or under, the supervision of a healthcare professional. The drug must be stored in a refrigerator when not in use. • Following the infusion there is a small chance you may experience an infusion-related reaction (discomfort, burning, swelling) and/or a reaction to the drug resulting dizziness or a rash
Oral treatment	• You have an inherited condition that may lead to you developing health problems. These problems could include tiredness, issues with your bones causing pain and becoming more likely to fracture, and enlargement of your liver and spleen which can result in serious complications. • In order to try and prevent problems developing you are required to receive treatment for the rest of your life. The treatment is effective as long as it is taken according to instructions. • The treatment you need to take is administered orally and can be taken with or without food. You need to take a capsule once to three times a day every day. The treatment does not require any special storage conditions. You also need to consider having your treatment with you when travelling. • Following the treatment you may experience a minor side-effect such as temporary diarrhea

### Valuation study

A sample of 100 members of the UK general public (aged 18+ and currently resident in the UK) were invited to participate in the valuation study through an established panel containing individuals who have given prior consent to be contacted for research studies, via a newspaper advertisement, and word-of-mouth. As per NICE requirements, the valuation study was performed with members from the UK general public [25]. The sample size of 100 is in line with what is used by other groups for similar studies [26, 27]. Participants were enrolled to approximate the UK general public as described by available census data with regard to age and gender [28]. Eligible individuals were to be aged 18 years of age or above and currently resident in the UK to be eligible. Participants in the study provided written informed consent, and demographic data were collected prior to the administration of the EQ-5D [29], a standard measure of health status. Face-to-face interviews were conducted by experienced interviewers following an interview guide specifically developed for this study.

During these face-to face interviews participants were first asked to read the various health states descriptions and rank them from ‘least preferred’ to ‘most preferred’ using a 100-point Visual Analogue Scale (VAS) running from 0 (‘worst imaginable health state’) to 100 (‘best imaginable health state’). Participants were also asked to rank a state called “dead”. This initial ranking exercise served as a warm up exercise and as an introduction to the concept of rating health states, a familiarisation with the descriptions and provided an indication about which states, if any, would typically be ranked as “worse than dead” [30].

During the TTO portion of the valuation study, a modified version of the Measurement and Valuation of Health (MVH) protocol was used [31]. Participants were first asked to rate health states that they had ranked as “better than dead” during the VAS exercise. During the TTO procedure participants were asked to imagine that they were in a selected health state for a 10-year long period. For each health state, participants could choose between remaining in the health state without improvement for ten years, or reducing the number of years of life to be lived in a state of full health. The process incorporated a ‘ping-pong’ approach with years traded back and forth between higher and lower values up to the point of indifference. A utility value for each health state was assigned depending on where this point occurred. A modified approach was undertaken with states ranked as “worse than dead” during the VAS exercise. In this instance the trade-off occurred between being ‘dead’ and spending time in the particular state followed by a period of full health. During the interview process, all health states were identified using symbols; no reference was made to the name of the health state, the condition or the treatment.

### Statistical analysis

The analysis population included all participants who were interviewed and who had met the inclusion criteria.

The collected sociodemographic and EQ-5D data, as well as TTO and VAS data were summarised using descriptive statistics. Quantitative (continuous) variables were described by their mean, standard deviation (SD), minimum and maximum values. Qualitative (categorical) variables were described by the frequency of each response choice. Missing items were not imputed.

All data processing and analyses were performed with SAS® software for Windows Version 9.2 or later (SAS Institute, Inc., Cary, NC, USA).

## Results

### Study participants

One hundred members of the UK general public participated in the valuation study. Demographic characteristics and EQ-5D scores of the study population are presented in Table 2. Mean age of the participants was 35 years, and 66% were women.

**Table 2:** Demographic characteristics and EQ-5D VAS and index scores in the study population (*n* = 100)

Characteristics	Study population (*n* = 100)
Age - years, mean (SD)	34.5 (10.2)
Gender - Female, n	66
Qualifications, n	
Left school with no qualifications	4
Left school with qualifications	7
Completed some college	18
Degree/postgraduate level	71
Main activity, n	
Employed full-time	66
Employed part-time	24
Student	2
Unemployed	3
Retired	1
Other	4
Marital status, n	
Single	35
Partnership	26
Married	33
Divorced/separated	6
Taking an oral treatment, n	
Yes / No	15 / 85
Receiving an intravenous treatment, n	
Yes / No	0 / 100
EQ-5D VAS score, mean (SD)	86.1 (10.5)
EQ-5D Index score, mean (SD)	0.95 (0.14)

*SD,* Standard deviation

Among the 100 participants, 15 were regularly taking some form of oral medication, and none were receiving a treatment intravenously.

### Valuation of the health states

Table 3 presents the mean TTO-derived health state values which are expressed on a scale from 0 (health state equivalent to being dead) to 1 (heath state equivalent to being in full health). For the “controlled disease” state, the mean VAS value was 71.4 and the mean TTO value was 0.89, indicating a good health state, which is around the mean EQ-5D single index score for the UK population of 0.86 [32] (Table 3). For the “intravenous treatment” state, the mean VAS value was 50.0 and the mean TTO value was 0.73. The observed decrement (− 0.16) in utility for this state compared to the “controlled disease” state was largely above the threshold that has been suggested by Feeny et al. (2005) as a meaningful difference [33]. It highlights the significant impact on HRQL linked to intravenous treatment. For the generic “oral treatment” state, there was only a marginal reduction in HRQL as compared to the “controlled disease” state with a mean VAS value of 71.0 and a mean TTO value of 0.85. The decrement of utilities observed between the “controlled disease” state and the generic “oral treatment” was below the aforementioned 0.05 threshold suggesting no meaningful difference. For the “oral treatment, alternative 1” state (reduced frequency of intake and side effects), the mean VAS value was 66.5 and the mean TTO value was 0.82. For the “oral treatment, alternative 2” state (higher frequency of intake and side effects), the mean VAS value was 59.1, and the mean TTO value was 0.78 (Table 3).

**Table 3:** Values from VAS and TTO exercises in the UK general public (*n* = 100)

Health states	VAS value(*N* = 100)	TTO value(*N* = 100)
Controlled disease		
Mean (SD)	71.4 (17.9)	0.89 (0.11)
Min - Max	10.0–98.0	0.45–1.00
Intravenous treatment		
Mean (SD)	50.0 (17.8)	0.73 (0.20)
Min - Max	0.0–90.0	0.05–1.00
Oral treatment		
Mean (SD)	71.0 (17.1)	0.85 (0.15)
Min - Max	10.0–96.0	0.25–1.00
Oral treatment, Alternative 1		
Mean (SD)	66.53 (17.49)	0.82 (0.23)
Min - Max	20.0–97.0	−0.85-1.00
Oral treatment, Alternative 2		
Mean (SD)	59.1 (19.4)	0.78 (0.18)
Min - Max	10.0–94.0	0.15–1.00

*SD,* Standard deviation

Oral treatment, alternative 1: reduced frequency of intake and side effects

Oral treatment, alternative 2: higher frequency of intake and more frequent occurrence of side effects

## Discussion

This study was specifically conducted to estimate the utility values associated with each health state related to treatment mode of administration in Gaucher disease, through the use of the TTO method with members of the general public in the UK.

The utility values elicited for the health state descriptions follow expected patterns with increased treatment mode of administration burden being associated with a subsequent decline in utility. Looking firstly at the generic mode of administration states, it is clear that the “controlled disease” state had the highest mean TTO value (0.89). This is not surprising as the description deliberately omits any mention of the burden associated with the administration mode. This does, however, serve as a benchmark for understanding the burden associated with particular treatment modes. The “oral treatment” state demonstrates only a marginal reduction in HRQL (0.85). This presents a credible depiction of the burden associated with the necessity for frequent oral medication for an indefinite period. A more marked impact is evident for the “intravenous treatment” state (0.73). A utility difference of − 0.12 between oral and intravenous treatments suggests this additional burden is not trivial and represents a genuine challenge to the preservation of HRQL for patients. The difference in utility between the mode of administration is in line with other studies comparing utilities for oral treatment and subcutaneous infusion for the management of iron overload [34, 35] or intravenous treatment for the maintenance treatment of cytomegalovirus retinitis in individuals with HIV [36]. As an example, in one of these studies, participants attributed a utility value of 0.85 to the once daily oral treatment of iron overload in the context of beta-thalassaemia while they attributed a lower value of 0.61 to the subcutaneous treatment consisting in 8–12 h long infusions for 5–7 days a week [34]. It is clear that in addition to the mode of administration itself, other factors may contribute to the magnitude of the difference in utility values between treatment options, including but not limited to factors such as frequency of administration, potential treatment-related side-effects. In their study, Sakamaki et al. using various approaches, including TTO, reported a larger difference in utility values between the orally administered anticancer agent (TTO-derived utility value of 0.90) and the conventional intravenous chemotherapy (TTO-derived utility value of 0.68) in the treatment of gastric cancer patients; however, the latter treatment was associated with more severe side effects, hospitalisation and confinement to bed for the administration, in contrast to the oral treatment associated with milder side effects and constraints [37].

Looking at the two alternative oral treatment options, it is clear that the treatment which is associated with the need for more frequent administration and a greater incidence of adverse events has lower utility (0.78 vs. 0.82). The 0.04 difference between treatment options provides an indication of the magnitude of burden and suggests a noticeable difference in HRQL impact for patients.

Our findings in Gaucher disease could also be relevant to other lysosomal storage diseases linked to enzyme deficiency and for which oral and intravenous form of therapy is available. A recent discrete choice experiment study exploring the value that people placed on the different features of treatments for Fabry disease showed that oral treatment was preferred significantly over intravenous treatment [38].

There are several limitations in this study that should be noted. First, compared to the UK general population [28] our sample had a slightly higher proportion of women (66% vs 51% in the UK general population) and was slightly younger with median age 31 vs. 39 years, respectively. Overall, study participants reported being in a very good state of health with high EQ-5D single index score of 0.95. A previous study in the UK found a mean single index score for the UK population of 0.86 with people below 45 years of age reporting decidedly higher values in the 0.91–0.94 range [32]. Our sample may also be overrepresented with highly educated participants.

Another limitation of the study that one could raise is the absence of direct input from patients with Gaucher disease in the development of the health states descriptions. However, the purpose of the present study was to focus on specific known attributes related to the modes of treatment administration rather than the disease itself. The exploration of utilities related to various disease states in GD1 was already published by Ganz et al. [39]. Still, attempts were made to ensure that the health state descriptions were as representative as possible, but we cannot exclude the possibility of having overlooked some issues of importance to patients.

## Conclusions

In conclusion, the utility values provided by this study demonstrate the HRQL impact of changes in treatment modes of administration and treatment characteristic profiles. The data suggests that these differences are significant and could potentially have important consequences for patients. Despite the study limitations, such data may be used to help support economic evaluations of future treatments for Gaucher disease.

## Additional file

Additional file 1: **Table S1.** Health state descriptions. (DOCX 17 kb)
