# Supplementary material for: A health state utility valuation study to assess the impact of treatment mode of administration in Gaucher disease
Source: Orphanet J Rare Dis. 2018 Sep 10;13:159. doi: 10.1186/s13023-018-0903-6 (PMC6131903; doi:10.1186/s13023-018-0903-6)
Supplement: Supplementary file 1 — Table S1. Health state descriptions. (DOCX 17 kb) [file 13023_2018_903_MOESM1_ESM.docx]

**Supplementary Material**

**Table S1. Health state descriptions**

| **Health states** | **Descriptions** |
| --- | --- |
| Controlled disease | - - You have an inherited condition that may lead to you developing health problems. These problems could include tiredness, issues with your bones causing pain and becoming more likely to fracture, and enlargement of your liver and spleen which can result in serious complications.   - In order to try and prevent problems developing you are required to receive treatment for the rest of your life. The treatment is effective as long as it is taken according to instructions.   - You need to take the treatment on a regular basis. You need to consider your access to treatment when travelling. |
| Intravenous treatment | - - You have an inherited condition that may lead to you developing health problems. These problems could include tiredness, issues with your bones causing pain and becoming more likely to fracture, and enlargement of your liver and spleen which can result in serious complications.   - In order to try and prevent problems developing you are required to receive treatment for the rest of your life. The treatment is effective as long as it is taken according to instructions.   - The treatment you need to take is administered intravenously. You need to receive a 1 to 2-hour infusion (directly into a vein) every 2 weeks. You also need to consider your access to treatment when travelling as the infusion must be administered by, or under, the supervision of a healthcare professional. The drug must be stored in a refrigerator when not in use.   - Following the infusion there is a small chance you may experience an infusion-related reaction (discomfort, burning, swelling) and/or a reaction to the drug resulting dizziness or a rash |
| Oral treatment | - - You have an inherited condition that may lead to you developing health problems. These problems could include tiredness, issues with your bones causing pain and becoming more likely to fracture, and enlargement of your liver and spleen which can result in serious complications.   - In order to try and prevent problems developing you are required to receive treatment for the rest of your life. The treatment is effective as long as it is taken according to instructions.   - The treatment you need to take is administered orally and can be taken with or without food. You need to take a capsule once to three times a day every day. The treatment does not require any special storage conditions. You also need to consider having your treatment with you when travelling.   - Following the treatment you may experience a minor side-effect such as temporary diarrhea |
| Oral treatment (Alternative 1 – reduced frequency of intake and side effects) | - - You have an inherited condition that may lead to you developing health problems. These problems could include tiredness, issues with your bones causing pain and becoming more likely to fracture, and enlargement of your liver and spleen which can result in serious complications.   - In order to try and prevent problems developing you are required to receive treatment for the rest of your life. The treatment is effective as long as it is taken according to instructions.   - The treatment you need to take is administered orally and can be taken with or without food. You need to take a capsule once or twice a day every day. The treatment does not require any special storage conditions. You need to consider having your treatment with you when travelling.   - Following the treatment you may experience minor side-effects such as temporary diarrhea, headache and tiredness |
| Oral treatment (Alternative 2 – Higher frequency of intake and more frequent occurrence of side effects) | - - You have an inherited condition that may lead to you developing health problems. These problems could include tiredness, issues with your bones causing pain and becoming more likely to fracture, and enlargement of your liver and spleen which can result in serious complications.   - In order to try and prevent problems developing you are required to receive treatment for the rest of your life. The treatment is effective as long as it is taken according to instructions.   - The treatment you need to take is administered orally and can be taken with or without food. You need to take a capsule three times a day. The treatment does not require any special storage conditions. You need to consider having your treatment with you when travelling.   - Following the treatment you may experience minor side-effects such as temporary diarrhea, flatulence, abdominal pain, weight loss and tremors |
